# Cellular Mechanisms of Photobiomodulation in Relation to HeLa Kyoto Tumor Cells Exposed to Ionizing Radiation

**DOI:** 10.3390/ijms26189197

**Published:** 2025-09-20

**Authors:** Anna V. Maslennikova, Artem O. Belotelov, Elena I. Cherkasova, Vladimir I. Yusupov, Ulyana A. Kononova, Natalia Yu. Shilyagina, Dmitry V. Skamnitsky

**Affiliations:** 1 Department of Oncology and Radiation Therapy, Privolzhsky Research Medical University, Minina and Pozharskogo Sq., 10/1, pl. 603005 Nizhny Novgorod, Russia; arteom.belotelow@yandex.ru; 2 Department of Biophysics, Institute of Biology and Biomedicine, National Research Lobachevsky State University of Nizhny Novgorod, Gagarin Avenue 23, pl. 603022 Nizhny Novgorod, Russia; cherkasova.el@yandex.ru (E.I.C.); usyakononova@gmail.com (U.A.K.); nat-lekanova@yandex.ru (N.Y.S.); 3 Institute of Photonic Technologies, National Research Center “Kurchatov Institute”, Akademika Kurchatova 1, pl. 123182 Moscow, Russia; iouss@yandex.ru; 4 Research Institute of Clinical Oncology, Nizhny Novgorod Regional Clinical Oncological Dispensary, Rodionova Street 190, pl. 603093 Nizhny Novgorod, Russia; skamnitskiy@gmail.com

**Keywords:** gamma irradiation, photobiomodulation, HeLa Kyoto cell line, low-intensity red light, mitochondrial membrane potential, radiation-induced mitotic arrest, apoptosis

## Abstract

During the clinical use of photobiomodulation (PBM) to manage radiotherapy-induced side effects, tumor tissue may be exposed to low-intensity laser light. Therefore, it is necessary to evaluate potential unintended PBM stimulation of tumor cells when PBM combines with ionizing radiation (IR). We investigated the effects of PBM (0.3 J/cm^2^) on the cell cycle, mitochondrial potential, and cell death of HeLa Kyoto cells, comparing pre-irradiation (PBM→IR) and post-irradiation (IR→PBM) exposure to 2 Gy, 4 Gy, and 6 Gy of ionizing radiation. PBM prior to IR induced the radiation-induced arrest in the G_2_/M phase of the cell cycle. PBM after IR resulted in a partial release of cells from the radiation-induced arrest in the G_0_/G_1_ phase, along with a decrease in the number of apoptotic cells and cells with depolarized mitochondrial membranes compared to samples treated with IR only. These findings provide a basis for further research into PBM timing to improve radiotherapy outcomes.

## 1. Introduction

Acute and late skin and mucosal toxicities, along with associated mucositis, are among the most significant side effects of radiotherapy or chemotherapy for head and neck and genitourinary cancers [1,2]. For more than 20 years, low-intensity laser/LED light (photobiomodulation, PBM) has been used to manage radiation-induced complications [3] due to its anti-inflammatory, immunocorrective, and analgesic effects, as well as its ability to promote cell proliferative activity [4,5,6].

Due to its increasing use in oncology care and a better understanding of the biologic mechanisms and clinical outcomes, it is important to document the safety of PBM use in oncology settings [7,8,9]. For a long time, it was believed that, due to the absence of a thermal effect, PBM did not have a stimulating effect on viable tumor cells and therefore did not carry risks. This has changed over the last decade, when modern studies have identified a large number of signaling pathways that are activated by PBM and are significantly associated with the formation of an aggressive tumor phenotype [10,11].

Direct investigation of the radio-modulatory effects of PBM on tumor response is limited. It is now proven that the effect of the combination of low-intensity laser light and ionizing radiation on tumor cells can vary widely and lead to diverse effects depending on the fluence, wavelength, and sequence of exposure (PBM and IR). Most data indicate that PBM exposure prior to IR does not have a protective effect in relation to tumor cells, and instead may reduce the number of viable tumor cells both at high [12,13,14] and low PBM fluences [15]. Much less research discusses the influence of PBM following IR on tumor cells. There is information about both the absence of the stimulating effect of PBM [16,17] and about an increase in the number of viable cells of HeLa Kyoto culture exposed to light with a wavelength λ = 635 nm and a fluence F = 0.3 J/cm^2^ one hour after gamma irradiation at doses of 2 Gy, 4 Gy, and 6 Gy [15]. Most studies dedicated to the mechanisms of low-intensity red light combined with IR range the PBM fluences from 5 J/cm^2^ to 50 J/cm^2^, while clinical guidelines for the treatment of radiation-induced mucositis recommend PBM with a fluence not exceeding 2 J/cm^2^ [18].

The state of the mitochondrial membrane, cell cycle, and cell death pathways is known to be among the main targets of PBM. These issues are discussed in the most detail in [13], where PBM with λ = 685 nm and “high” fluences (5 J/cm^2^, 10 J/cm^2^, and 20 J/cm^2^) were used. This study showed a decrease in the number of viable cells when PBM was performed before IR. An increase in the number of apoptotic tumor cells was detected, as well as an increase in autophagy occurring with PBM at a fluence of 20 J/cm^2^. The work did not demonstrate changes in the cell cycle of tumor cells during exposure to IR in combination with PBM. There is information [19,20] that the loss of mitochondrial inner membrane potential is directly related to the early stages of apoptosis, coincides with the disruption of mitochondrial membrane permeability, and leads to the release of cytochrome-C into the cytosol, triggering subsequent events in the apoptotic cascade [21]. Assessment of the intrinsic mitochondrial transmembrane potential provides an opportunity to capture the early cellular response to combined exposure to IR and PBM, and to note the presence of a mitochondrial pathway for apoptosis induction.

The potential for PBM to negatively influence tumor growth and/or reaction to cytotoxic treatment has had a limited evaluation to date and is currently unresolved. Conflicting data refute or support the potential for PBM to impact tumor activity and responsiveness to treatment. Our study aimed to highlight the effects of PBM with a wavelength of 640 nm and ‘low’ fluence (less than 1 J/cm^2^) in combination with ionizing radiation on the cell cycle and membrane potential of HeLa Kyoto tumor cells, depending on IR dose and the sequence of the two exposures (PBM before or after IR).

## 2. Results

### 2.1. Viability of HeLa Kyoto Tumor Cells After IR

**Figure 1:**
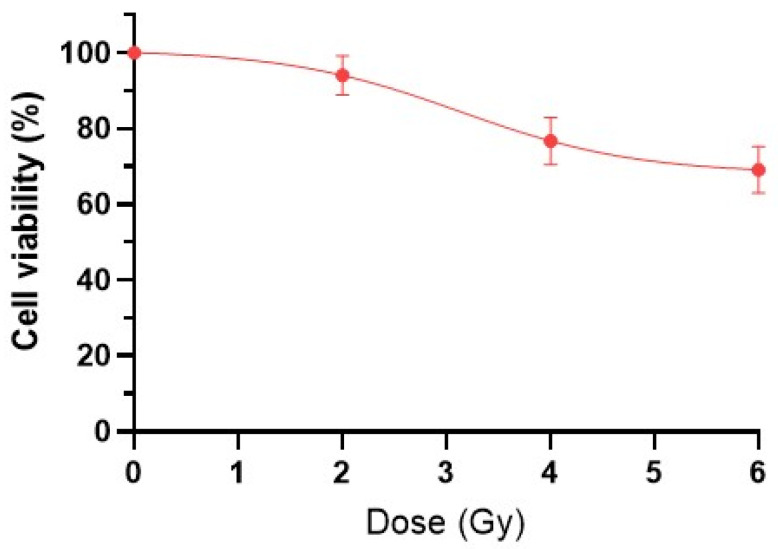
Viability of HeLa Kyoto cancer cells one day after irradiation with IR at doses of 2 Gy, 4 Gy, and 6 Gy.

To assess the effects of ionizing radiation on cell viability, two independent methods were employed: the MTT assay and colony formation analysis. Irradiation of HeLa Kyoto tumor cells showed a standard survival curve for the IR doses (2 Gy, 4 Gy, 6 Gy) at 24 h post-exposure (Figure 1).

The characteristic shoulder on the survival curve at a dose of 2 Gy is clearly visible in Figure 1, indicating the cells’ ability to repair radiation damage. With an increase in IR dose, there is a sharp decrease in the number of viable tumor cells to 79.7 ± 5.3% for 4 Gy and 69.1 ± 6.1% for 6 Gy, compared to the control (100 ± 5.4%).

### 2.2. Influence of Photobiomodulation on the Cell Cycle of HeLa Kyoto Cells

Analysis of the distribution of HeLa Kyoto culture cells across phases of the cell cycle when exposed only to low-intensity light without IR showed that PBM itself does not cause its changes.

**Figure 2:**
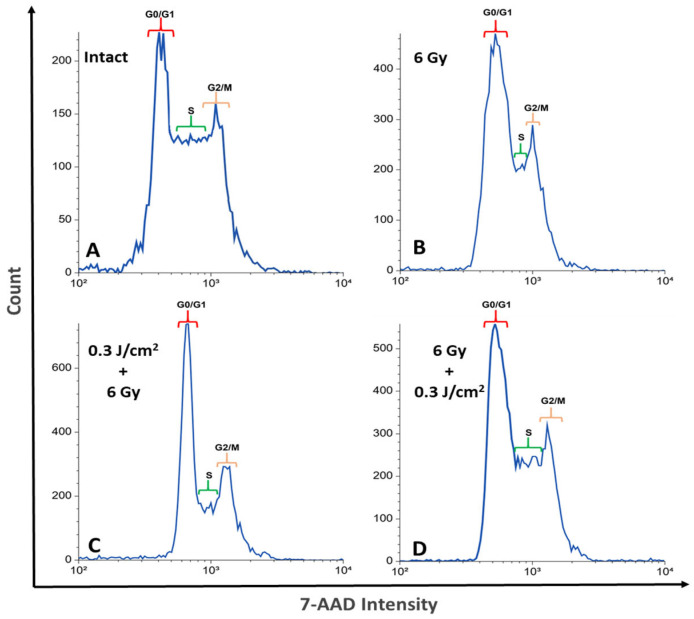
DNA content distribution across cell cycle phases: intact sample (**A**); after exposure to 6 Gy (**B**); 0.3 J/cm^2^ + 6 Gy (**C**); 6 Gy + 0.3 J/cm^2^ (**D**). For flow cytometric analysis, 10,000 events were recorded per sample.

Figure 2 shows an example of a DNA content histogram representing the distribution of cells across different cell cycle phases (G_0_/G_1_, S, G_2_/M). (A); after exposure to a dose of 6 Gy (B); after additional exposure to PBM before (C) and after (D) irradiation with IR. Supplementary Figure S1 shows the distribution of cell populations visualized using dot plots.

**Figure 3:**
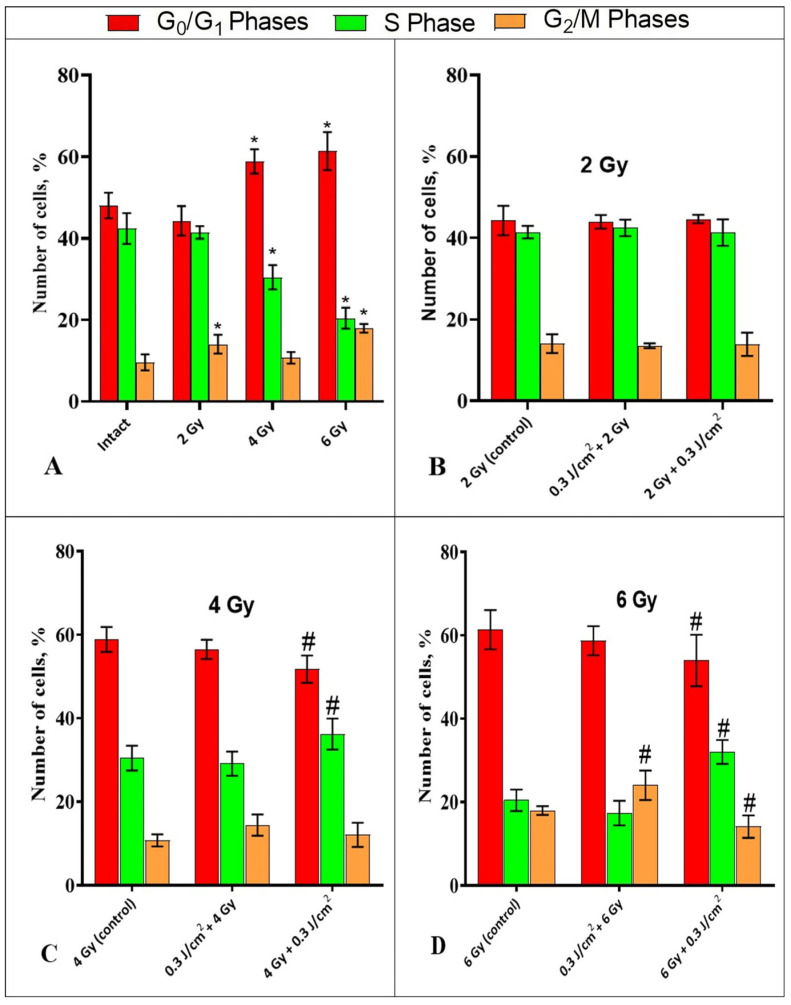
Distribution of HeLa Kyoto cells by phases of the cell cycle following experimental treatments. (**A**)—IR at a dose of 2 Gy, 4 Gy, and 6 Gy, compared with an intact sample. (**B**–**D**)—after combinations of “PBM→IR” and “IR→PBM” compared with irradiation at doses 2 Gy, 4 Gy, and 6 Gy, respectively, without PBM. Data are mean ±SD (n = 5 biological replicates). *—statistically significant differences relative to the “intact” group, *p* < 0.05. #—statistically significant differences relative to the “control” group, *p* < 0.05.

IR at a dose of 2 Gy did not cause any changes in the number of cells in G_0_/G_1_ and S phase, while a statistically significant increase from 9.6 ± 2% to 14 ± 2.3% in the number of cells in the G_2_/M phase was observed compared to intact cells (Figure 3A). Exposure to IR at doses of 4 Gy and 6 Gy led to a decrease in the number of cells in the S phase to 30.4 ± 3.2% and 20.4 ± 2.9%, respectively, compared to 42.4 ± 4% in the intact sample. Additionally, there was an increase in the number of cells in the G_0_/G_1_ phases of the cell cycle to 58.9 ± 3.3% and 61.3 ± 4.9% for doses of 4 Gy and 6 Gy, respectively, compared to 48 ± 3.4% in the intact sample. After exposure to a dose of 6 Gy, a statistically significant increase in the number of cells in the G_2_/M phases from 9.6 ± 2% to 17.9 ± 1.4% was observed, compared to the intact sample (Figure 3A). These changes can be considered as a manifestation of the typical cell response to irradiation—radiation-induced arrest.

The combined effect of PBM with F = 0.3 J/cm^2^ and IR at a dose of 2 Gy did not cause any changes in the ratio of cell cycle phases compared to cells irradiated with a dose of 2 Gy, regardless of the sequence of the two factors (Figure 3B). IR at a dose of 4 Gy in combination with PBM (IR→PBM) led to a significant decrease in the proportion of cells in G_0_/G_1_ phases from 58.9 ± 3.2% to 51.7 ± 3.6%, and an increase in the proportion of cells in S phase from 30.4 ± 3.1% to 36.2 ± 3.8%, compared to the control (4 Gy), with no changes in the number of cells in G_2_/M phase (Figure 3C).

Irradiation at a dose of 6 Gy in combination with PBM (IR→PBM) led to a substantial decrease in the proportion of cells from 61.3 ± 5% to 53.9 ± 6.2% for G_0_/G_1_ phase, and from 17.9 ± 1.2% to 14.1 ± 2.8% for G_2_/M phase, compared to the control group (6 Gy). There was also a significant increase in the proportion of cells in S phase from 20.4 ± 2.7% to 32 ± 2.8%, compared to the “6 Gy control” group (Figure 3D). When exposed at a dose of 6 Gy in combination with PBM (PBM→IR), there was an increase in the number of cells in G_2_/M phase from 17.9 ± 1.2% to 24 ± 3.7%, compared to cells exposed to a dose of 6 Gy (“control”), with no statistically significant changes in the proportion of cells in S and G_0_/G_1_ phases relative to the same group (6 Gy) (Figure 3D).

Thus, PBM after gamma irradiation contributed to partial relief of radiation mitotic arrest; PBM before irradiation, on the contrary, contributed to the delay in cell division in the G_2_/M phase when exposed to a dose of 6 Gy.

### 2.3. The Study of the Impact of PBM in Combination with IR on the Process of Cell Death of HeLa Cells

**Figure 4:**
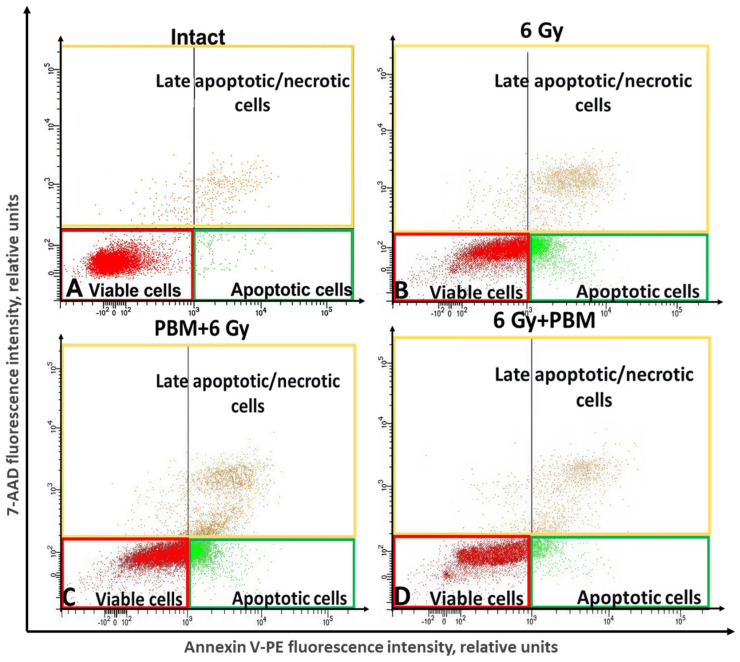
An example of the distribution of populations into late apoptotic/necrotic, early apoptotic, and living cells. Intact sample (**A**); after exposure to 6 Gy (**B**); with complex exposure 0.3 J/cm^2^ + 6 Gy (**C**); and 6 Gy + 0.3 J/cm^2^ (**D**). For flow cytometric analysis, 10,000 events were recorded per sample.

Figure 4 shows examples of cell population distribution into late apoptotic/necrotic, early apoptotic, and living cells in an intact sample, after irradiation with a dose of 6 Gy in combination with PBM before and after gamma irradiation.

**Figure 5:**
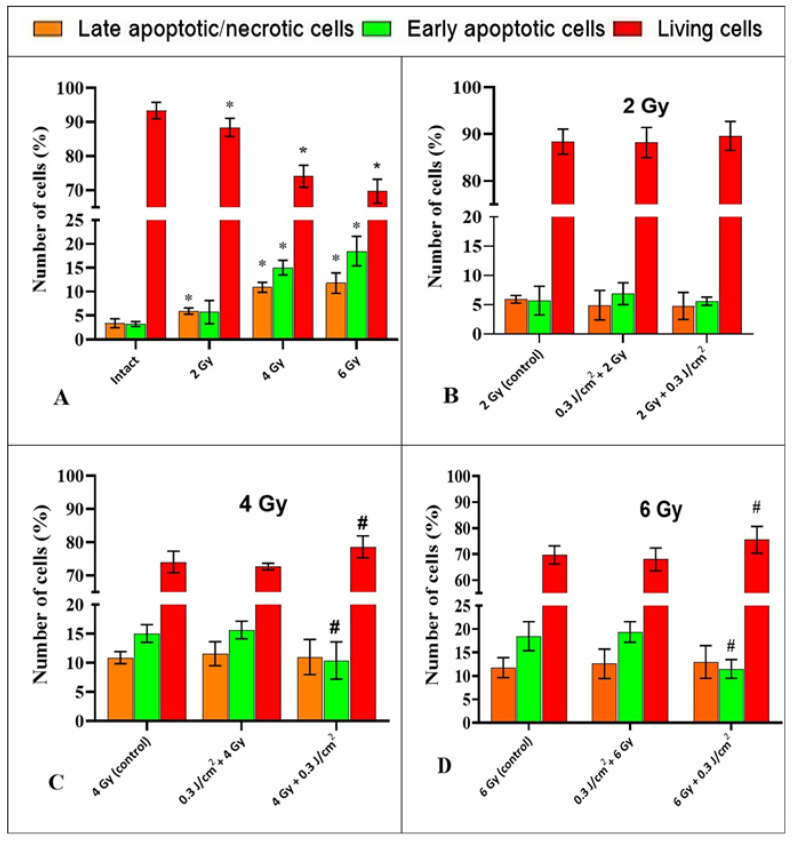
The ratio of late apoptotic/necrotic, early apoptotic, and living HeLa cells following experimental treatments. (**A**)—exposure to IR in different doses compared to an intact sample. (**B**–**D**)—effects of combinations of “PBM→IR” and “IR→PBM” compared with the corresponding control (2 Gy, 4 Gy, and 6 Gy without PBM). Data are mean ±SD (n = 4 biological replicates). *—statistically significant differences relative to the “intact” group, *p* < 0.05. #—statistically significant differences relative to the “control” group, *p* < 0.05.

First, the influence of ionizing radiation without PBM on the process of cell death of HeLa tumor cells was investigated. In the intact sample, the percentage of late apoptotic/necrotic cells and cells entering the apoptotic pathway was 3.4 ± 0.9% and 3.2 ± 0.6%, respectively. Exposure to gamma radiation at a dose of 2 Gy resulted in a decrease in the number of living cells in the irradiated sample to 88.4 ± 3%, with the percentage of late apoptotic/necrotic cells and cells entering the apoptotic pathway increasing to 5.9 ± 0.7% and 5.7 ± 2.4%, respectively, compared to the intact sample (Figure 5A). Exposure to IR at a dose of 4 Gy led to an increase in the populations of late apoptotic/necrotic cells and cells in the early apoptotic state to 10.9 ± 1.1% and 15 ± 1.6%, as well as a decrease in the number of living cells to 74 ± 3.3%, compared to the intact sample (Figure 5A). After irradiating cells at a dose of 6 Gy, an increase in the number of late apoptotic/necrotic and early apoptotic cells to 11.8 ± 2.3% and 18.5 ± 3.1% was also observed, with a decrease in the number of living cells to 69.7 ± 1.9%, compared to the intact sample, respectively (Figure 5A).

The combined effects of IR at a dose of 2 Gy and PBM did not lead to significant changes in the distribution of HeLa cells into early apoptotic, living, and late apoptotic/necrotic cells compared to cells exposed to IR alone (2 Gy), regardless of the sequence of the two interventions (Figure 5B). After exposure to “4 Gy + 0.3 J/cm^2^,” the proportion of early apoptotic cells decreased to 10.4 ± 2.7%, compared to the control (15 ± 1.6%), irradiated only with IR; this decrease was statistically significant (Figure 5C). The number of late apoptotic/necrotic cells in the “4 Gy + 0.3 J/cm^2^” group did not change significantly, while the number of living cells increased from 74 ± 3.3% to 78.6 ± 3.6%, compared to the “4 Gy control” group (Figure 5C). When studying the combined effects of “6 Gy + 0.3 J/cm^2^” on HeLa cells, a statistically significant decrease in the proportion of early apoptotic cells to 11.5 ± 2.1%, compared to the “6 Gy control” (18.5 ± 3.1%), was observed. The number of late apoptotic/necrotic cells in this group remained unchanged, while the population of viable cells increased to 75.5 ± 5.2% compared to the control (69.7 ± 1.9%) (Figure 5D). The differences were statistically significant. After exposure to “0.3 J/cm^2^ + 4 Gy,” the ratio of living cells, late apoptotic/necrotic cells, and cells in the apoptotic state remained practically unchanged compared to the “4 Gy” group (Figure 5C). Similarly, after exposure to “0.3 J/cm^2^ + 6 Gy,” the ratio remained unchanged compared to the “6 Gy” group (Figure 5D).

### 2.4. The Study of the Impact of PBM in Combination with IR on the Mitochondrial Membrane Potential of HeLa Kyoto Cells

**Figure 6:**
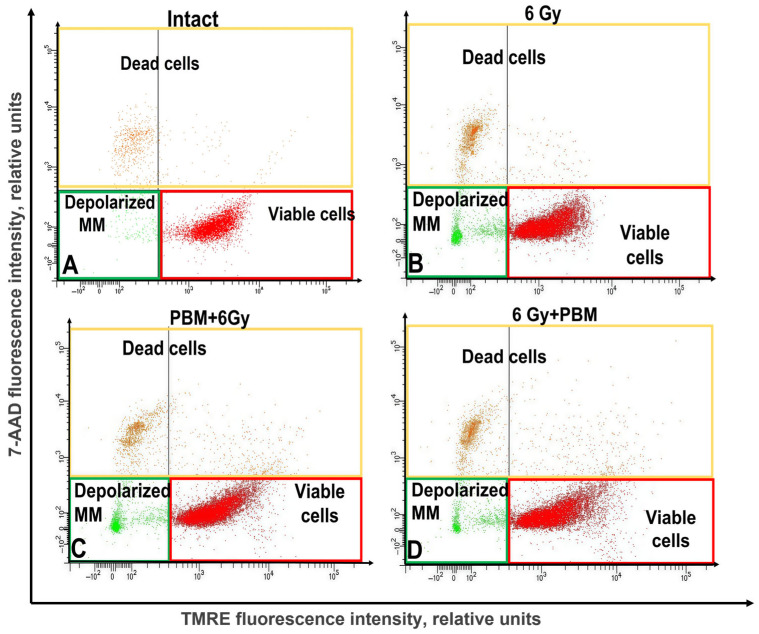
An example of cell distribution across populations: viable, dead, and with a depolarized mitochondrial membrane. Intact sample (**A**); after exposure to 6 Gy (**B**); with complex exposure 0.3 J/cm^2^ + 6 Gy (**C**); and 6 Gy + 0.3 J/cm^2^ (**D**). For flow cytometric analysis, 10,000 events were recorded per sample.

Figure 6 shows examples of cell population distribution into living cells, dead cells, and cells with depolarized mitochondrial membrane in an intact sample, after irradiation with a dose of 6 Gy in combination with PBM before and after gamma irradiation.

IR leads to an accumulation of cells in the lower left part of the image compared to the intact sample, corresponding to cells with depolarized mitochondrial membrane. After 6 Gy + 0.3 J/cm^2^ exposure, a decrease in the population with depolarized mitochondrial membrane, compared to the control group “6 Gy,” is observed.

**Figure 7:**
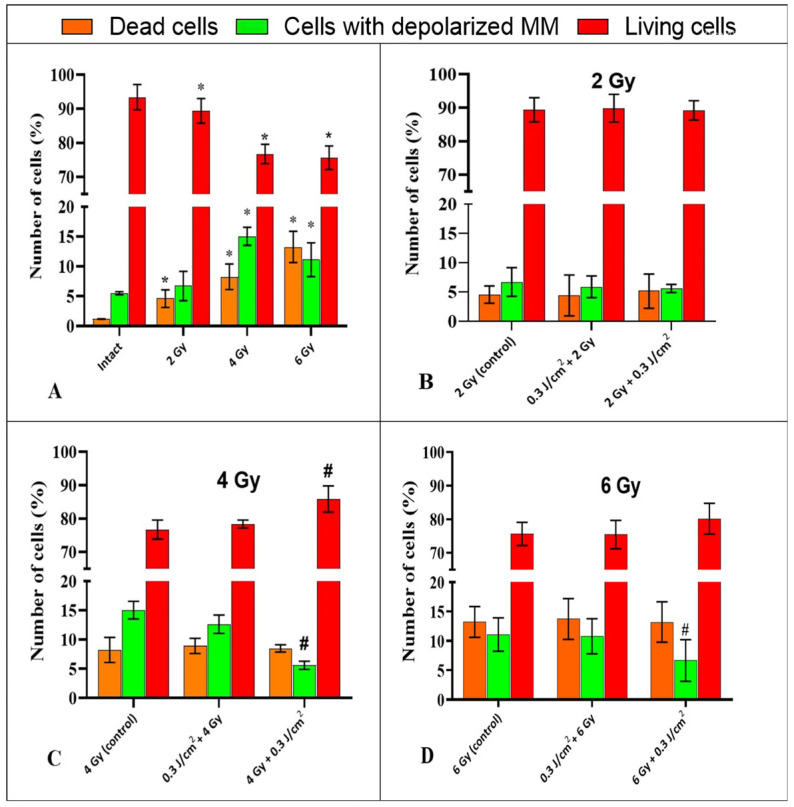
The ratio of viable, dead, and depolarized cells (MM) following experimental treatments. (**A**)—exposure to IR in different doses compared to an intact sample. (**B**–**D**)—combined effects in combinations of “PBM→IR” and “IR→PBM” compared with the corresponding control without PBM. Data are mean ±SD (n = 4 biological replicates). *—statistically significant differences relative to the “intact” group, *p* < 0.05. #—statistically significant differences relative to the “control” group, *p* < 0.05.

The energy exchange in the cell and its physiological state directly depend on the condition of the mitochondria, which is determined by the integrity of their inner membrane and the transmembrane potential formed on it [22]. In our study, it was found that in intact samples, the population of living HeLa Kyoto cells with intact inner mitochondrial membrane (MM) was 93.4 ± 3.3%, the population of dead cells with intact MM was 1.2 ± 0.2%, and the population of cells with depolarized MM was 5.5 ± 0.5%. This distribution indicates a good condition of the cell culture, the absence of pathological processes, and an adequate response to reagent treatment (Figure 7A). Irradiation at a dose of 2 Gy resulted in an increase in the proportion of dead cells to 4.6 ± 1.5% and a decrease in the population of living cells to 89.4 ± 3.9% compared to the intact sample. The number of cells with depolarized MM remained virtually unchanged (Figure 7B). Irradiation at a dose of 4 Gy led to a decrease in the number of living cells with intact membrane to 76.7 ± 2.8%, and an increase in the number of dead cells and cells with depolarized mitochondrial membrane to 8.2 ± 2.1% and 15 ± 1.6%, respectively, compared to the intact control (Figure 7C). Exposure to IR at a dose of 6 Gy resulted in a decrease in the number of viable cells to 75.7 ± 3.6%, an increase in the number of dead cells to 13.2 ± 2.8%, and cells with depolarized MM to 11.1 ± 3% relative to the intact sample (Figure 7D).

PBM in combination with irradiation at a dose of 2 Gy did not lead to significant changes in the mitochondrial potential of HeLa cells compared to cells exposed to IR alone (2 Gy), regardless of the sequence of the two exposures (Figure 7B). After exposure to “4 Gy + 0.3 J/cm^2^”, the percentage of cells with depolarized mitochondrial membrane significantly decreased to 5.6 ± 0.9%, compared to cells after exposure to IR alone (Figure 7C). The number of dead cells in the “4 Gy + 0.3 J/cm^2^” group did not change significantly, while the number of living cells increased significantly from 76.7 ± 2.8% to 85.8 ± 4.2%, compared to the “4 Gy control” group (Figure 7C). When studying the combined effect of “6 Gy + 0.3 J/cm^2^” on HeLa cells, a statistically significant decrease in the percentage of cells with depolarized mitochondrial membrane to 6.7 ± 3.5%, compared to cells exposed to “6 Gy,” was observed (Figure 7D). The number of dead cells in this group did not change, while the population of living cells increased to 80.1 ± 4.8% compared to the control. The differences were statistically significant. After exposure to “0.3 J/cm^2^ + 4 Gy”, the ratio of living cells, dead cells, and cells with depolarized mitochondrial membrane remained almost unchanged compared to the “4 Gy” group (Figure 7C), as well as after exposure to “0.3 J/cm^2^ + 6 Gy” (Figure 7D).

## 3. Discussion

The aim of our study was to investigate the mechanisms of cellular response in tumor HeLa cells to low-intensity light (F < 1 J/cm^2^) and IR radiation at clinically relevant doses, depending on the sequence of these two exposures.

A typical cellular response to ionizing radiation involves cell cycle arrest—particularly a radiation-induced mitotic arrest—which provides time for error correction and DNA damage repair [23]. Such division delays can occur during both G_0_/G_1_ and G_2_/M phases of the cell cycle [22]. When exposed to gamma radiation only, we observed a statistically significant increase in the number of cells in the G_2_/M phase after irradiation at doses of 2 Gy and 6 Gy, and an increase in the number of cells in the G_0_/G_1_ phase after irradiation at doses of 4 Gy and 6 Gy, with a corresponding decrease in the number of cells in the S phase.

The combination of 6 Gy + 0.3 J/cm^2^ resulted in a decrease in cell numbers in the G_0_/G_1_ and G_2_/M phases, with an increase in the S-phase population compared to the IR-only irradiated control (Figure 3D). With a similar exposure at a dose of 4 Gy, there was a decrease in the number of cells in the G_0_/G_1_ phase, and an increase in the proportion of cells in the S phase, compared to gamma irradiation alone (Figure 3C). Based on this, it can be assumed that PBM contributes to the partial release of the radiation-induced mitotic arrest and passage through the G_0_/G_1_ cycle control point, resulting in cell transition to the S phase. This corresponds to an increase in the number of viable tumor cells after exposure to IR at doses of 4 Gy and 6 Gy and subsequent PBM [15], and is consistent with [16], which showed an increase in the proportion of cells in the S phase and a decrease in the proportion of cells in the G_1_ phase after exposure to IR at doses of 2.5 Gy and 10 Gy and PBM in the fluence range of 30 J/cm^2^ to 150 J/cm^2^. It can be suggested that the increase in S-phase cell numbers is associated with more efficient DNA repair under PBM influence; however, we cannot state this definitively. It is possible that this increase results from aberrant progression—a process enabling cells with altered DNA to bypass checkpoints and advance through the cell cycle [24]. This requires further investigation using a comprehensive set of DNA status assessment methods to clarify this issue.

The combination “0.3 J/cm^2^ + 6 Gy” resulted in an increase in the number of cells in the G_2_/M phase from 17.9 ± 1.2% to 24 ± 3.7% compared to cells exposed to a dose of 6 Gy (“control”), without statistically significant changes in the proportion of cells in the S phase and G_0_/G_1_ phase (Figure 3D). Accordingly, it can be assumed that PBM before IR initiates mechanisms that prevent cell cycle checkpoint release, resulting in a statistically significant decrease in the number of viable tumor cells after exposure in this mode [15]. This result corresponds to [25], where PBM before IR caused an increase in the G_2_/M cells by 27% in 24 h after irradiation.

One of the important cellular targets for IR is the electron transport chain of mitochondria [26,27,28]. IR exposure can disrupt the electron transport chain, resulting in the restoration of transport enzymes and a decrease in the mitochondrial membrane potential, as well as an increase in the concentration of reactive oxygen species in the cell [29]. Low-intensity light exposure can reactivate carrier enzymes in the mitochondrial respiratory chain, restoring electron flow and forming a transmembrane potential, leading to the creation of a proton concentration gradient with subsequent ATP production, formation of ROS, and further activation of numerous signaling pathways aimed at restoring normal cell function [30,31]. The initiation of a cellular response to red light PBM is believed to occur due to the photoactivation of endogenous cellular chromophores. In the case of red light, such a candidate may be the IV complex of the electron transport chain in mitochondria—cytochrome C oxidase [32,33].

Our research revealed a significant decrease in the number of cells with depolarized mitochondrial membrane and an increase in the number of cells with undamaged MM when using PBM after 4 Gy and 6 Gy exposure, compared to cells after gamma irradiation alone. These results are in line with previous findings showing an increase in the number of viable cells in the MTT assay when exposed to IR→PBM [15]. Discussing the mechanisms of this phenomenon, it can be assumed that PBM after radiotherapy contributes to the activation of protective pathways in cells damaged by IR. This is consistent with research results that have demonstrated the stimulatory effect of PBM specifically on damaged cells [34].

At the same time, we did not reveal changes in the number of cells with depolarized mitochondrial membrane in the samples exposed to low-intensity red light before IR. These results correspond to our previous data on the absence of a protective effect of PBM on tumor cells, and to research data that showed a decrease in the mitochondrial activity of tumor cells upon exposure to PBM with λ = 780 nm and λ = 660 nm at an energy density of F = 1–6 J/cm^2^ [35]. These results may indicate that PBM is not able to trigger compensatory mechanisms in the mitochondria of intact tumor cells and does not protect them from IR.

There is evidence that PBM with fluences of 0.3 J/cm^2^ and 3 J/cm^2^ at λ = 808 nm without additional IR intervention leads to an increase in the number of cells undergoing apoptosis [36]. In other studies, when using red light laser radiation with F = 10–60 J/cm^2^, an enhancement of apoptosis was also observed through the activation of various signaling pathways [37]. Our study showed that PBM (0.3 J/cm^2^) after IR leads to a decrease in the number of early apoptotic tumor cells compared to samples after IR alone. This result corresponds to a reduction in the number of cells with depolarized mitochondrial membranes after IR + PBM. It can be assumed that PBM with a low fluence (0.3 J/cm^2^), by affecting mitochondrial function, can restore the transmembrane potential, thereby mitigating the consequences of radiation-induced mitochondrial damage.

## 4. Materials and Methods

### 4.1. Cell Line

We used the conditionally “immortal” human cervical carcinoma cell line HeLa Kyoto (Shemyakin-Ovchinnikov Institute of Bioorganic Chemistry, RAS (IBCh RAS), Moscow, Russia) as the subject of research. The cells were cultured under aseptic conditions in a 25 cm^2^ culture flask in a culture medium containing 90% DMEM nutrient medium (PanEco, Moscow, Russia), 10% embryonic bovine serum (HyClone, São Paulo, Brazil), and 2 mM L-glutamine (PanEco, Moscow, Russia). The cells were incubated and cultivated at 37 °C and 5% CO_2_ atmosphere in a cell incubator. Cell detachment for seeding in the experiment was carried out using a 0.25% trypsin-EDTA solution with Hank’s salts (PanEco, Moscow, Russia). During the experiment, the cells were counted in a Goryaev counting chamber and seeded in 25 cm^2^ culture flasks (300 thousand cells per flask at seeding). The cells were incubated throughout the experiment without changing the nutrient medium and cultivation conditions.

### 4.2. Assessment of Cell Radiosensitivity

The radiosensitivity of the HeLa Kyoto cell line to ionizing radiation was evaluated after irradiation at doses of 0, 2, 4, and 6 Gy. Quantitative analysis was performed using the MTT assay in three biological replicates, after which the obtained data were confirmed by the colony formation assay method in one replicate, which demonstrated results consistent with the MTT assay data.

MTT assay. Cells were seeded into 96-well plates (2000 cells/well) and incubated for 24 h at 37 °C and 5% CO_2_. After 24 h, the cultures were subjected to remote irradiation using a Novalis TX linear accelerator (Varian Medical Systems, Palo Alto, CA, USA) (doses: 0, 2, 4, and 6 Gy; dose rate 6 Gy/min; 6 MV mode). After irradiation, the cells were incubated for an additional 24 h, then MTT solution was added at a 1:10 ratio to the volume of the culture medium and incubated for 3 h at 37 °C. The formed formazan crystals were dissolved in 100 μL of DMSO (“PanEco”, Moscow, Russia), and the optical density was measured at a wavelength of 570 nm using a Synergy Mx plate reader (Biotek Instruments, Inc., Winooski, VT, USA). The results were normalized to the unirradiated control.

To confirm the MTT assay results, a colony formation assay was additionally performed. Cells were seeded into 6-well culture plates at a density of 200 cells/well and incubated for 24 h at 37 °C and 5% CO_2_. Cell irradiation was carried out under identical conditions (doses: 0, 2, 4, and 6 Gy; Novalis TX; 6 MV; 6 Gy/min), after which they were incubated for 10 days. After the incubation period, the cells were fixed with 70% ethanol, stained with a 0.1% methylene blue solution, and colonies containing more than 50 cells were counted. The survival fraction (SF) was calculated as the ratio of the plating efficiency in the experimental groups to that of the unirradiated control.

### 4.3. Photobiomodulation

The fluence of 0.3 J/cm^2^ was selected to evaluate the effects of PBM combined with IR on mitochondrial potential, cell cycle alterations, and cell death.

The cell culture flask was illuminated with low-intensity red light at a wavelength of λ = 640 ± 11 nm for 75 s. The light source used for PBM was the ‘CDM-08’ (Photon Technologies Institute, Moscow, Russia), which is based on an LED matrix with light passing through a lens of 4 cm diameter. The beam divergence angle was 60 degrees. The light intensity at the working surface was monitored using a FieldMaster power meter with an LM-10HTD sensor head (Coherent, Santa Clara, CA, USA). To analyze the spectral composition, a USB4000 spectrometer (Ocean Optics, Largo, FL, USA) with a resolution of 1.5 nm was employed. The distance from the lens to the surface of the flask, positioned under the emitter, was 46 cm, ensuring uniform illumination with an intensity of 4 ± 0.5 mW/cm^2^. The PBM parameters are summarized in Table 1.

### 4.4. Ionizing Radiation

Cells were irradiated using a Novalis Tx linear accelerator (Varian Medical Systems, Palo Alto, CA, USA, beam energy 6 MV, dose rate 6 Gy/min). Radiation exposure was performed once at clinically relevant doses (2 Gy, 4 Gy, and 6 Gy). To achieve a uniform dose distribution inside the culture flask due to the peculiarities of the depth distribution of the high-energy photon beam, cells were irradiated from below, with the beam passing through 5 cm plates made of water-equivalent polystyrene “RW3”. Calculations of irradiation parameters and dose distribution were performed in the Eclipse dosimetric planning system using the AcurosXB 16.1 calculation algorithm based on CT images of the culture flask.

### 4.5. Experimental Design

First, we examined the effect of PBM on tumor cells pre-exposed to IR (IR→PBM) (Supplementary Figure S2A). For experiments on a flow cytometer, cells were seeded onto a culture flask with an area of 25 cm^2^ at a density of 300 thousand cells per flask, and incubated without changing the medium for 24 h. After the incubation period, the attached cells on the culture flasks were irradiated with doses of 2 Gy, 4 Gy, and 6 Gy, and one hour later, PBM was performed with the parameters indicated in the table (Table 1). After PBM, the cells were incubated, then removed from the culture flasks and transferred to test tubes for flow cytometry, where the cells were additionally processed with dyes according to staining protocols. The effect of PBM on mitochondrial membrane potential (MM), cell death, and cell cycle was analyzed 24 h after PBM. Control cells irradiated with doses of 2 Gy, 4 Gy, and 6 Gy without PBM were used.

The effect of PBM on tumor cells pre-exposed to low-intensity light and then exposed to IR (PBM→IR) (Supplementary Figure S2B) was studied with the same cell seeding and incubation parameters and flow cytometry staining protocols. After 24 h of incubation following seeding, the cells were exposed to low-intensity light, and one hour later, gamma irradiation was performed with doses of 2 Gy, 4 Gy, and 6 Gy. The mechanisms of PBM action on the cells were analyzed 24 h after IR irradiation. Control cells irradiated with doses of 2 Gy, 4 Gy, and 6 Gy without PBM were used. For all flow cytometry experiments, we used a FACSAia III cytometer (Becton, Dickinson and Company, Erembodegem, Belgium); data acquisition and analysis were performed using FACSDiva™ software (Becton, Dickinson and Company, v. 7.0, Franklin Lakes, NJ, USA). 

### 4.6. Cell Cycle Definition

Cell cycle phase distribution (G_0_/G_1_, S, G2/M) was assessed in five independent biological experiments with two technical replicates each. The percentage of cells in each phase was calculated relative to the total number of cells in the analyzed population. The assessment of the combined effect of PBM→IR and IR→PBM on the cell cycle of HeLa Kyoto cells was carried out using flow cytometry with the APC BrdU Flow Kit set (Cat. No. 552598; Becton, Dickinson and Company, Franklin Lakes, NJ, USA), including bromodeoxyuridine (BrdU), Fluorochrome-conjugated anti-BrdU antibodies (APC), and 7-aminoactinomycin (7-AAD). Cells stained with both dyes, BrdU and 7-AAD, were used as positive controls, while non-stained cells were used as negative controls. Gating was performed based on the presence of BrdU and 7-AAD dyes in cells: cells in G_0_/G_1_ phases: BrdU (−), 7-AAD (−); cells in S phase: BrdU (+), 7-AAD (−), and BrdU (+), 7-AAD (+); cells in G_2_/M phases: BrdU (−), 7-AAD (+).

Cell preparation, BrdU cell labeling, subsequent staining, flow cytometric analysis, and interpretation of the results were conducted according to the BD Pharmingen™ BrdU Flow Kits Instruction Manual.

### 4.7. Transmembrane Mitochondrial Potential Study

The percentages of live cells, dead cells, and cells with depolarized mitochondrial membrane potential were determined in five independent biological experiments with two technical replicates each. Calculations were performed relative to the total number of cells within the defined analysis gate.

The processing for determining the mitochondrial potential was carried out according to the manufacturer’s protocol (BD Pharmingen™, San Diego, CA, USA). Staining of cells with TMRE (BD Pharmingen, San Diego, CA, USA) was carried out simultaneously with staining with the fluorescent dye 7-AAD (7-aminoactinomycin D, cat № 559763, BD Biosciences, San Jose, CA, USA), which allows assessment of the integrity of the cell’s plasma membrane. 7-AAD penetrates cells through damaged membrane and binds to fragmented/degraded DNA at sites between guanine and cytosine, which is a marker of late-stage apoptosis or necrosis [38]. This combination of dyes also allows dividing the cell population into living (positive for TMRE and negative for 7-AAD) cells, cells with depolarized mitochondrial membrane or “damaged” cells (negative for both dyes), and dead/dying cells (negative for TMRE and positive for 7-AAD). According to the instructions, gating was performed based on the presence of these dye groups in the cells.

### 4.8. Assessment of Cell Death Pathways

The percentage distribution of late apoptotic/necrotic, early apoptotic, and living cells was evaluated in three independent biological experiments with two technical replicates each. Percentages were determined relative to the total number of cells. Cell death assessment in cell culture was performed using the Annexin V-Phycoerythrin (PE) Apoptosis Detection Kit (cat. no. 559763, Becton, Dickinson and Company, Franklin Lakes, NJ, USA). The use of two dyes, Annexin V and 7-AAD, allowed for the discrimination of three distinct cell populations: (1) live cells (Annexin V^−^/7-AAD^−^); (2) early apoptotic cells (Annexin V^+^/7-AAD^−^); (3) late apoptotic/necrotic cells (Annexin V^+^/7-AAD^+^). This kit allows for the quantitative determination of apoptotic cells in a population [39].

Sample preparation, cell staining with annexin-V PE and 7-AAD, cytometric analysis, and interpretation of the results were conducted in accordance with the Technical Data Sheet of the PE Annexin V Apoptosis Detection Kit.

### 4.9. Statistics

Cell cycle analysis, mitochondrial transmembrane potential changes, and cell death assessment following PBM→IR and IR→PBM exposures were performed in at least three independent biological replicates with two technical replicates each.

Experimental data were statistically analyzed using GraphPad Prism 8.4.3 software (GraphPad Prism Software Inc., San Diego, CA, USA). The Kruskal–Wallis test was applied to evaluate the statistical significance of differences in mean values compared to control samples, with a significance threshold of *p* < 0.05. The exact number of replicates for each specific analysis is provided in the corresponding method subsections.

## 5. Conclusions

Photobiomodulation demonstrates multidirectional effects on tumor cells exposed to ionizing radiation, depending on the sequence of the two exposures. The “IR→PBM” protocol results in a decrease in cells with depolarized mitochondrial membrane and a partial removal of radiation-induced mitotic arrest of HeLa Kyoto cells. These changes correlate with an increase in the number of viable tumor cells after exposure to a PBM fluence of 300 mJ/cm^2^. Conversely, the “PBM→IR” sequence leads to an increase in the number of cells in the G_2_/M phase, which indicates the realization of mechanisms preventing effective DNA repair after the damaging effect. Thus, the state of mitochondria (cell energetics) and DNA repair systems are among the key targets of PBM in relation to irradiated cells. Molecular mechanisms of the above effects require further investigation.

## Figures and Tables

**Table 1:** PBM parameters.

Device	Fluence, J/cm^2^	Distance from the Emitter, cm	Intensity, mW/cm^2^	Time, s
CDM-08	300 ± 38	46	4.0 ± 0.5	75

## Data Availability

All data are included in the manuscript and Supplementary Materials.

## Supplementary Materials

The following supporting information can be downloaded at: https://www.mdpi.com/article/10.3390/ijms26189197/s1.
