# Supplementary material for: Cellular Mechanisms of Photobiomodulation in Relation to HeLa Kyoto Tumor Cells Exposed to Ionizing Radiation
Source: Int J Mol Sci. 2025 Sep 20;26(18):9197. doi: 10.3390/ijms26189197 (PMC12470394; doi:10.3390/ijms26189197)

International Journal of Molecular Sciences

Supplementary Materials for:

Cellular mechanisms of photobiomodulation in relation to HeLa Kyoto tumor cells exposed to ionizing radiation

Anna V. Maslennikova [1f*], Artem O. Belotelov [1,2a], Elena I. Cherkasova [2b], Vladimir I. Yusupov [3c], Ulyana A. Kononova [2d], Natalia Yu. Shilyagina [2e], Dmitry V. Skamnitsky [4g]

[1]Dept. of Oncology and Radiation Therapy, Privolzhsky Research Medical University, Minina and Pozharskogo Sq., 10/1, pl. 603005, Nizhny Novgorod, Russia.
[2]Institute of Biology and Biomedicine, National Research Lobachevsky State University of Nizhny Novgorod, Gagarin Avenue 23, pl. 603022, Nizhny Novgorod, Russia.
[3] National Research Centre «Kurchatov Institute», Akademika Kurchatova 1, pl. 123182, Moscow, Russia.
[4]Research Institute of Clinical Oncology, Nizhny Novgorod Regional Clinical Oncological Dispensary, Rodionova Street 190, pl. 603093, Nizhny Novgorod, Russia.
*arteom.belotelow@yandex.ru
[b]e-mail: cherkasova.el@yandex.ru
[c]e-mail: iouss@yandex.ru
[d]e-mail: usyakononova@gmail.com
[e]e-mail: nat-lekanova@yandex.ru
[f]e-mail: maslennikova.anna@gmail.com-corresponding author
[g]e-mail: skamnitskiy@gmail.com

Table of Contents

Item Number	Description	Page
Figure S1	An example of the distribution of cell populations by phases of the cell cycle under various treatments.	2
Figure S2	Design of an experiment to study the stimulating and adaptive effects of PBM in combination with IR on Hela Kyoto cells.	3

Figure S1. An example of the distribution of cell populations by phases of the cell cycle: intact sample (A); after exposure: 6 Gy (B); 0.3 J/cm² + 6 Gy (C); 6 Gy + 0.3 J/cm² (D).

The influences used did not result in significant visible redistributions within the three populations considered: the location of the analyzed groups remained the same, but the number of events in the regions of interest has changed.

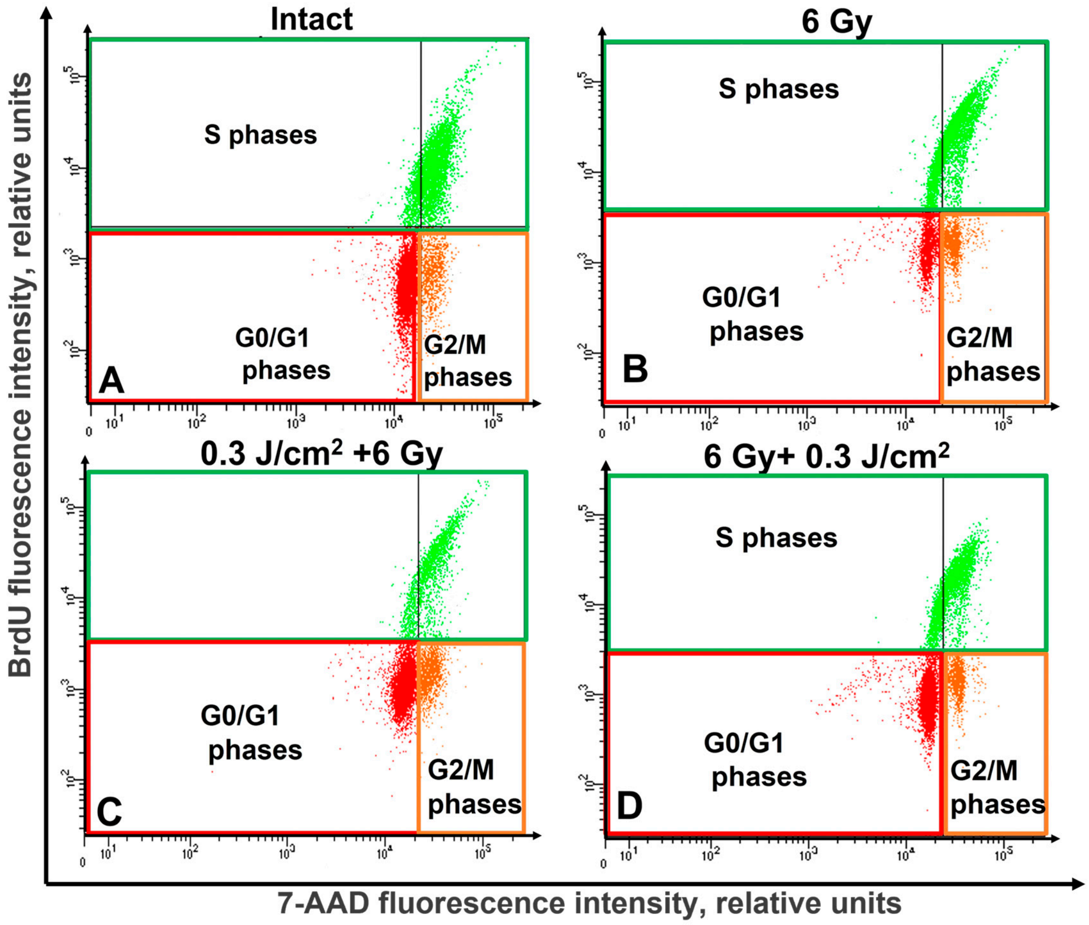

Figure S2. Design of an experiment to study the stimulating (PBM after IR) (A) and adaptive (PBM before IR) (B) effect of PBM in combination with the effects of IR on Hela Kyoto cells.

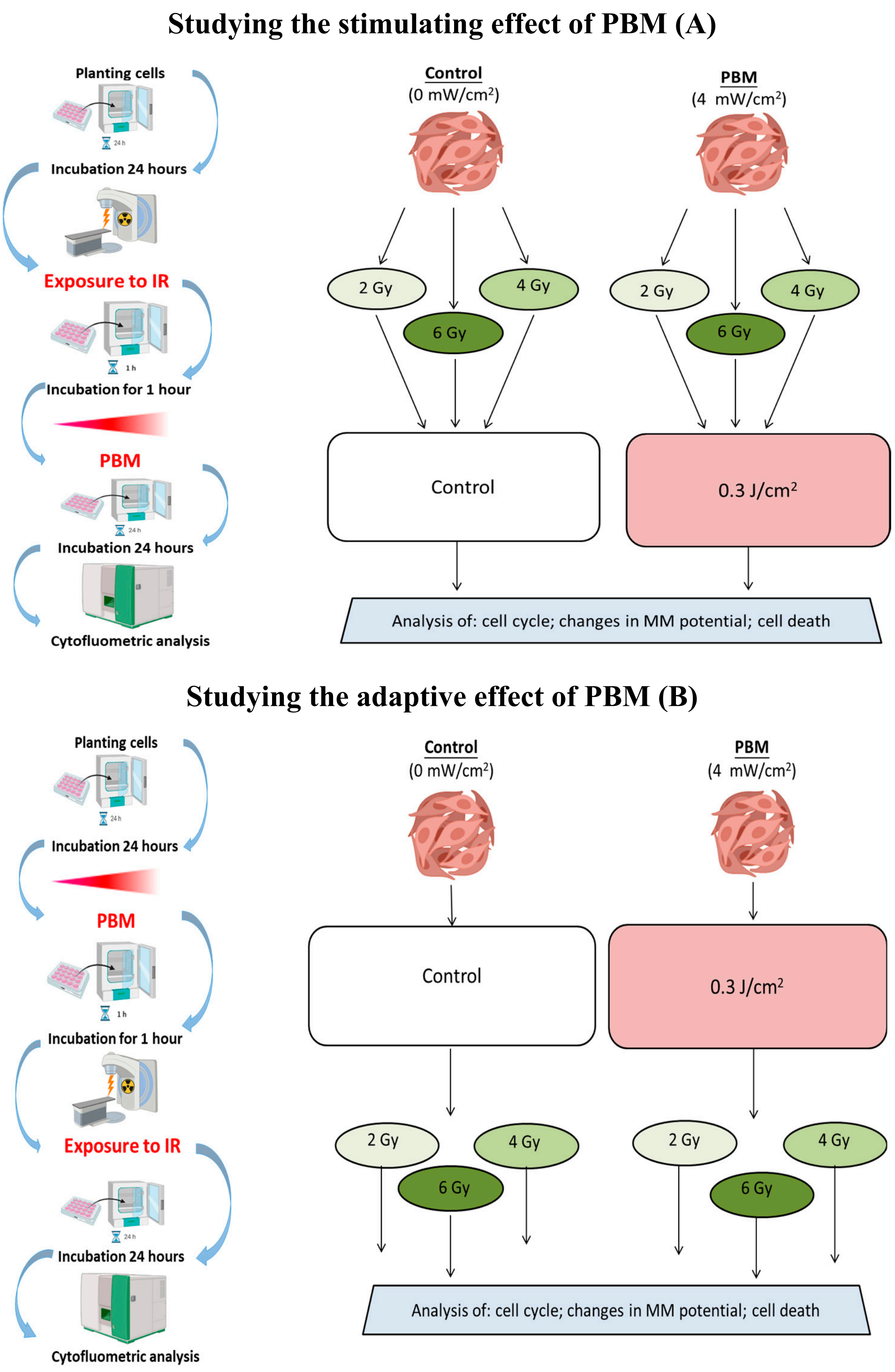

Supplement: Supplementary file 1 [file ijms-26-09197-s001.zip › ijms-3802829-supplementary.pdf]
